# Links between Vitamin D Deficiency and Cardiovascular Diseases

**DOI:** 10.1155/2015/109275

**Published:** 2015-04-27

**Authors:** Ioana Mozos, Otilia Marginean

**Affiliations:** ^1^Department of Functional Sciences, “Victor Babes” University of Medicine and Pharmacy, 300173 Timisoara, Romania; ^2^Department of Pediatrics, “Victor Babes” University of Medicine and Pharmacy, 300173 Timisoara, Romania

## Abstract

The aim of the present paper was to review the most important mechanisms explaining the possible association of vitamin D deficiency and cardiovascular diseases, focusing on recent experimental and clinical data. Low vitamin D levels favor atherosclerosis enabling vascular inflammation, endothelial dysfunction, formation of foam cells, and proliferation of smooth muscle cells. The antihypertensive properties of vitamin D include suppression of the renin-angiotensin-aldosterone system, renoprotective effects, direct effects on endothelial cells and calcium metabolism, inhibition of growth of vascular smooth muscle cells, prevention of secondary hyperparathyroidism, and beneficial effects on cardiovascular risk factors. Vitamin D is also involved in glycemic control, lipid metabolism, insulin secretion, and sensitivity, explaining the association between vitamin D deficiency and metabolic syndrome. Vitamin D deficit was associated in some studies with the number of affected coronary arteries, postinfarction complications, inflammatory cytokines and cardiac remodeling in patients with myocardial infarction, direct electromechanical effects and inflammation in atrial fibrillation, and neuroprotective effects in stroke. In peripheral arterial disease, vitamin D status was related to the decline of the functional performance, severity, atherosclerosis and inflammatory markers, arterial stiffness, vascular calcifications, and arterial aging. Vitamin D supplementation should further consider additional factors, such as phosphates, parathormone, renin, and fibroblast growth factor 23 levels.

## 1. Introduction

Vitamin D exists in two forms: D2 (ergocalciferol) and D3 (cholecalciferol). Vitamin D3, “the sunshine vitamin,” is synthetized in the human epidermis via ultraviolet irradiation, or it may be consumed in the form of oily fish or supplements. Vitamin D2 is found in plants, as a product of irradiation of ergosterol [1]. The vitamin is converted in the liver and kidney to calcidiol and calcitriol, respectively, and acts on specific target tissues via vitamin D receptors. Calcitriol, the active form of vitamin D, binds to vitamin D receptors in the intestines, bones, and kidneys to increase calcium absorption from the intestines, promote calcium deposition in bones, and decrease parathyroid hormone concentrations (PTH). Its extraosseous effects are less known. Vitamin D receptors were found in other tissues, as well, including the brain, cardiomyocytes, vascular smooth muscle cells, endothelial cells, pancreatic beta-cells, skeletal muscle, breast, prostate, colon, macrophages, and skin, exerting several pleiotropic effects, and their expression decreases with age. The vitamin D receptor is closely related to the thyroid, retinoid, and peroxisome proliferator-activator receptors [2]. Recent studies have found active 1 alpha hydroxylase in several extra renal tissues, such as the heart and vascular smooth muscle cells [3–5]. Activated vitamin D may influence cellular growth, proliferation and apoptosis, oxidative stress, membrane transport, matrix homeostasis, cell adhesion, and immune system functions and may regulate a large number of genes and healthy aging [6, 7].

Vitamin D insufficiency is a common public health problem, very often unrecognized and untreated, associated with rickets, dental caries, and growth retardation in children and osteomalacia, osteopenia, osteoporosis, decreased muscle strength, falls, and increased risk of fracture in adults. Vitamin D insufficiency is associated with indoor lifestyle, sun avoidance strategies, obesity, diabetes mellitus, low HDL cholesterol, older age, distance from the equator, darker skin, winter season, air pollution, smoking, malabsorption, renal and liver disease, and medication (anticonvulsants, glucocorticoids, antirejection, and human immunodeficiency virus therapy) [1–11]. The biologically active form of vitamin D is 1,25 dihydroxyvitamin D, but the best indicator of vitamin D status in individuals free of kidney disease is 25-hydroxyvitamin D, the substrate for the renal and nonrenal production of calcitriol, with a longer biological half-life and a higher concentration than 1,25 dihydroxyvitamin D, reflecting the total endogenous and exogenous production of vitamin D [12, 13].

Recent research has linked inadequate vitamin D status to nonskeletal major chronic diseases, especially cardiovascular diseases [8]. Existing data from laboratory studies, epidemiologic and experimental research and prevention trials, suggest that vitamin D reduces the risk of cardiovascular disease, and a large, randomized, primary prevention trial, with adequate dosing, combining cholecalciferol and omega-3 fatty acids, is ongoing: the VITAL study. Poor vitamin D status was associated with cardiovascular and overall mortality, despite unconvincing results of vitamin D supplementation on mortality [13]. Food-based strategies for enhancement of vitamin D status in the population could lower cardiovascular risk if a causal link between low vitamin status and cardiovascular pathology would be demonstrated [14].

The aim of the present paper was to review the most important mechanisms explaining the possible association of vitamin D deficiency and cardiovascular diseases, focusing on recent experimental and clinical data.

## 2. Definition of Vitamin D Deficiency

Optimal serum concentration of 25-hydroxyvitamin D considers only bone health and was defined as the concentration that maximally suppresses serum parathyroid hormone [15]. Most experts define vitamin D deficiency as a calcidiol level of <20 ng/mL and insufficiency as 21–29 ng/mL [1, 16]. Vitamin D is sufficient if >30 ng/mL, and vitamin D intoxication is considered if >150 ng/mL [16]. There are variations among professional bodies regarding the cut-off values for insufficient or deficient vitamin D level [17].

According to a report of the Institute of Medicine (IOM), vitamin D at doses of 600 IU/day is beneficial for the bones, but it is not certain if higher doses could reduce the risk of chronic diseases, including cancer and cardiovascular pathology [17]. A threshold effect between vitamin D status and cardiovascular risk was suggested [11]. Zittermann et al. found a vitamin D level of 30–35 ng/L as the best choice for risk reduction in cardiovascular mortality [18].

## 3. Vitamin D and Renin-Angiotensin-Aldosterone System

The renin-angiotensin-aldosterone system (RAA) maintains vascular resistance, due to angiotensin II synthesis, and extracellular fluid volume homeostasis, considering the release of aldosterone [19]. Studies by different groups, in both animals and humans, demonstrated that vitamin D* decreases RAA activity* [20], suppressing* renin gene expression* [12]. Vitamin D regulates the genes involved in renin production, through a* cis*-DNA element in the renin gene promoter [1, 21], downregulating the RAA system.

Vitamin D receptor-null mice had a sustained elevation of renin expression, maintaining a normal level of blood electrolytes [19]. Increased renin synthesis leads to an elevated angiotensin II production, which is a strong vasoconstrictor, enabling the development of hypertension and left ventricular hypertrophy [19]. Similarly, 1-alpha-hydroxylase deficient mice, unable to synthetize the active metabolite 1,25-dihydroxyvitamin D, develop also high blood pressure and left ventricular hypertrophy [4]. Studies using renal arteries from hypertensive patients reported that calcitriol* reduces the expression of the angiotensin-1 receptor in endothelial cells,* improving endothelial function and preventing reactive oxygen species overproduction [22]. Secondary* hyperparathyroidism* in vitamin D receptor-null mice may also contribute to renin upregulation [19], considering that intravenous infusion of PTH increases plasma renin activity and renin release [23, 24].

Vitamin D regulation of renin expression is independent of calcium metabolism, and calcitriol markedly suppresses renin transcription by a vitamin D receptor—mediated mechanism in cell cultures [19]. Ferder et al. suggested a possible feedback link between vitamin D and the renin-angiotensin system (RAS), considering that vitamin D and angiotensin II receptors are distributed in the same tissues, changes in RAA activity and activation of the vitamin D receptors seem inversely related, and vitamin D deficiency could be explained by the cellular* inflammatory response activity* induced by the RAA system [25]. Therapy should combine RAS blockade and VDR stimulation [25].

D hypervitaminosis induces vascular and soft-tissue calcifications.* Calcium deposition in the vascular smooth muscle cells* may also lead to RAA activation [26–28].

Suppression of renin production and downregulation of the RAA may explain the direct myocardial and vascular effects through* modulation of hypertrophic stimuli* [10].

## 4. Vitamin D and Atherosclerosis

Vitamin D suppresses inflammation via several pathways, such as inhibition of prostaglandin and cyclooxygenase pathways, upregulation of anti-inflammatory cytokines, decrease of cytokine induced expression of adhesion molecules, reduction of matrix metalloproteinase 9, and downregulation of the RAA [11, 25]. Vitamin D deficiency stimulates* systemic and vascular inflammation,* enabling atherogenesis [1]. On the other hand, as already mentioned, hypertension is also associated with lack of vitamin D, due to activation of the RAA system, enabling* endothelial dysfunction*, the first step in plaque formation. The proinflammatory nuclear factor kB mediates partly the association between endothelial dysfunction and low vitamin D status [11].

Large epidemiological studies have highlighted vitamin D deficiency as a marker of cardiovascular risk [29], promoting accelerated atherosclerosis [12, 30] and subsequent cardiovascular events [11]. Chronic vitamin D deficiency causes* secondary hyperparathyroidism, increasing insulin resistance*, impairing beta-pancreatic cell function, and enabling the development of metabolic syndrome and diabetes mellitus [1]. Calcitriol regulates the genes involved in insulin production in the pancreas [1].

Vitamin D has also some* antiatherogenic functions*, inhibiting the formation of foam cells, cholesterol uptake by the macrophages, and enabling HDL transport [31]. Lower serum 25-hydroxyvitamin D was associated with the metabolic syndrome and its components, especially HDL cholesterol concentration [32].

Vascular smooth muscle cells and endothelial cells express receptors for vitamin D, enabling conversion of calcidiol to calcitriol [12], and vitamin D is involved in* regulation of growth and proliferation of smooth muscle cells and cardiomyocytes* [12, 33, 34]. Vitamin D inhibits proliferation of vascular smooth muscle cells by acute influx of calcium into the cell and increases calcification of smooth muscle cells [34].

The cardiovascular protective effects of vitamin D include also the anti-inflammatory effects, inhibition of vascular smooth muscle cells proliferation, suppression of proatherogenic T lymphocytes, preservation of endothelial function [22, 35–40], and protection against advanced glycation products [2].

Vitamin D deficiency was associated with* vascular stiffness*, which is a known predictor of cardiovascular morbidity and mortality [11] and a marker of subclinical atherosclerosis.

## 5. Vitamin D Insufficiency and Hypertension

Low vitamin D levels have been associated with increased prevalence of hypertension [41–43] or elevated diastolic blood pressure [44–46]. Clinical studies demonstrated an* inverse, dose-response relationship* between plasma 1,25(OH)2D3 concentration and blood pressure or* renin activity* in both normotensive and hypertensive patients [43, 47–49]. Wang et al. reported associations of hypertension risk and plasma 25-hydroxyvitamin D and vitamin D receptor polymorphism, respectively [8]. Rats with experimentally induced vitamin D deficiency developed hypertension and cardiomegaly [43, 50]. Mice lacking vitamin D receptor had an increased renin expression and angiotensin II production and developed also hypertension and cardiac hypertrophy [19, 43, 51]. Lower end-diastolic pressures were noted in Dahl salt-sensitive rats treated with paricalcitol, a vitamin D receptor activator, compared to untreated animals [52].

Ultraviolet light exposure enables vitamin D synthesis and has blood pressure lowering effects [53, 54]. Hypertensive patients exposed to a tanning bed significantly raised their concentration of 25-hydroxyvitamin D after 3 months and became normotensive [54]. Vitamin D3 supplementation reduces blood pressure in patients with essential hypertension [55, 56] and reduces also plasma renin activity and angiotensin II levels in hyperparathyroidism patients [57, 58]. Erythemal and preerythemal doses of UV irradiation decrease vascular resistance, with diffuse skin vasodilatation, related to nitric oxide release [2, 59].

Calcitriol exerts a protective effect on human renovascular function, restoring the impaired* endothelium-dependent relaxation* in renal arteries, accompanied by the normalization of oxidative-stress related proteins [22]. The augmented production of* reactive oxygen species* is induced by angiotensin II in human renal arteries and endothelial cells and impairs vascular function enabling the development of hypertension [22]. Vitamin D metabolites reduced endothelium-dependent vascular smooth muscle contractions and vascular tone in hypertensive rats by affecting calcium influx across endothelial cells [60].* In vitro*, vitamin D receptor activation induces a concentration-dependent increase of nitric oxide production in endothelial cells and improves the angiogenic properties of endothelial progenitor cells [61, 62].

Hypertensive patients with vitamin D deficiency were associated with a twofold risk of cardiovascular events, including myocardial infarction, angina, prolonged chest pain with documented ECG changes, stroke, transient ischemic attack, peripheral claudication, and heart failure [12].

Not all studies demonstrated blood pressure lowering effects of vitamin D. High-dose intermittent vitamin D therapy was given every 2 months in patients with resistant hypertension, on, at least, 3 antihypertensive agents, for 6 months, with no reduction of office blood pressure, 24-hour ambulatory blood pressure, or left ventricular mass [63].

The relation of vitamin D deficiency and* preeclampsia* is controversial. Vitamin D deficiency in pregnancy has been associated with an increased risk of preeclampsia [64, 65], suggesting that vitamin D supplementation in early pregnancy could prevent preeclampsia [64] and decreased calcidiol was found at diagnosis of early onset severe preeclampsia [66]. On the other hand, several authors reported no adverse pregnancy outcomes, including preeclampsia, with low 25-hydroxyvitamin D [67, 68]. An inverse association was reported between calcium intake and maternal blood pressure, as well, as the incidence of preeclampsia syndrome, explained, probably, by the influence on parathyroid hormone release and intracellular calcium availability, but the relationship between calcium and risk of hypertension in pregnancy seems to be inconsistent and inconclusive [69]. Preeclampsia is associated with reduced placental perfusion and maternal endothelial dysfunction [62]. Vitamin D increases capillary formation in endothelial colony forming cells, probably mediated by increased expression of* vascular endothelial growth factor and promatrix metalloproteinase activity* [62].

The mode of* vitamin D prophylaxis during infancy* (continuous daily supplementation, bolus doses of vitamin D forte every three months during the first year of life, or bolus doses during winter combined with continuous daily drops during summer) did not influence the blood pressure level in early adolescence, and no adverse effects were reported despite exceeding daily doses [70].

Vitamin D levels were significantly lower in patients with* orthostatic hypotension*, but lower vitamin D status was not associated with impaired orthostatic hemodynamics [46]. Vitamin D deficiency may also be involved in orthostatic hypotension development in elderly patients [71]. Serum levels of vitamin D should be checked during the evaluation of those patients, considering that orthostatic hypotension is associated with falls, fractures, cardiovascular events, and significant mortality in the elderly [46, 71]. Vitamin D receptors are found in vascular smooth muscle, endothelial and cardiac cells, enabling involvement in the cardiac and vascular response during orthostasis [72], and vitamin D deficiency downregulates the RAA system and was also associated with endothelial dysfunction [46]. On the other hand, both orthostatic hypotension and vitamin D deficiency are more prevalent in the elderly.

Women's Health Initiative study of* postmenopausal women* found no reduction in blood pressure with supplementation of 1,000 mg/day of calcium and 400 IU/day of vitamin D3, probably because the vitamin dose was insufficient for beneficial blood pressure effects [73].

*The parathormone (PTH)* is a crucial regulator of calcium and phosphate balance. Higher PTH concentrations were associated with several cardiovascular risk factors, including hypertension and arterial stiffness [74]. The mechanisms linking PTH and blood pressure include upregulation of RAA due to increase of serum calcium and sympathetic activity [74]. PTH correlated with blood pressure and hypertension incidence in a cross-sectional study, including 1,205 elderly subjects; serum vitamin D was not associated with blood pressure, probably due to the relatively high levels in the study population [75].

The antihypertensive properties of vitamin D include suppression of the RAA system, renoprotective effects including antiproteinuric and anti-inflammatory effects, direct effects on endothelial cells and calcium metabolism, inhibition of growth of vascular smooth muscle cells, prevention of secondary hyperparathyroidism, and beneficial effects on common cardiovascular risk factors and are important in vitamin D deficient, hypertensive patients [42, 44, 76]. Observational studies suggest that vitamin D deficiency is associated with high blood pressure, but randomized clinical trials did not yield conclusive results [20].

## 6. Vitamin D and Metabolic Syndrome

Cardiovascular risk factors such as hyperlipidemia, abdominal obesity, hypertension, and diabetes often cluster in the same individual [42]. A small cross-sectional study, including middle-aged men, found that vitamin D metabolites were related to lipid and glucose metabolism and serum urate [77]. Serum calcitriol was inversely correlated to the blood pressure and triglycerides and calcidiol to fasting insulin and lipoprotein lipase activity both in adiposal tissue and in skeletal muscle [77].

Statins exert beneficial effects, not only on lowering cholesterol but also on diabetes, bone metabolism, and inflammatory states, probably related to vitamin D [78]. Atorvastatin used in patients with acute coronary syndromes decreased cholesterol levels and significantly increased vitamin D levels, due to the shared metabolic pathway of cholesterol and vitamin D [42, 79].

Obese individuals are at higher risk of hypertension, hypercholesterolemia, diabetes, cardiovascular mortality, and vitamin D deficiency. Besides vitamin D sequestration in subcutaneous fat and making stores less available to become biologically activated, obese persons may have a sedentary lifestyle and be less active outdoors, and skin production of vitamin D may be impaired due to clothing habits [42, 80].

Many cellular, experimental, and observational studies support the role of vitamin D in the pathogenesis of type 1 and type 2 diabetes [81]. Type 1 and type 2 diabetic patients have a higher incidence of hypovitaminosis D [81]. A lower incidence of type 2 diabetes was found after vitamin D supplementation in high risk individuals, supporting the hypothesis that high vitamin D status protects against type 2 diabetes [82]. Insulitis and type 1 diabetes mellitus were prevented by pharmacologic doses of vitamin D in nonobese mice, possibly by immune modulation and direct effect on beta-cell function [81]. An inverse association between circulating 25-hydroxyvitamin D and incident type 2 diabetes was demonstrated by a systematic review and meta-analysis, including only prospective studies [83]. The plausible mechanisms explaining the mentioned associations involve vitamin D receptors in pancreatic beta-cells influencing insulin secretion and vitamin D effects on insulin sensitivity, or through the effects of vitamin D on calcium metabolism, but there is no demonstrable evidence of causality yet [27, 83, 84]. Sun exposure implies greater outdoor physical activity, improving insulin sensitivity [84]. Further mechanism connecting vitamin D and diabetes mellitus involves pancreatic tissue and cells of the immune system expressing not only vitamin D receptors but also vitamin D binding protein, some allelic gene variations involving vitamin D metabolism and vitamin D receptors, associated with glucose intolerance, insulin secretion and sensitivity, and inflammation [81]. Thus, vitamin D has an important role in glycemic control, which may influence cardiovascular outcomes [27].

Vitamin D may influence several components of the metabolic syndrome, especially hypertension, hyperglycemia, insulin resistance, and hyperlipidemia.

## 7. Vitamin D, Coronary Heart Disease, and Heart Failure

A strong association was found between vitamin D deficiency and* slow coronary flow, endothelial dysfunction and subclinical atherosclerosis,* in patients with normal or near-normal coronary arteries at coronary angiography [85]. Decreased levels of vitamin D binding protein were found in the plasma of survivors of a myocardial infarction at young age, statistically correlated with the number of affected coronary arteries [86]. Low vitamin D levels have been linked to inflammation,* higher coronary artery calcium scores, increased mean platelet volume,* and* increased vascular stiffness* [11, 28]. Abnormally high mean platelet volume has been associated with cardiovascular diseases, considering the higher risk to block arteries, due to the ability to aggregate more rapidly with collagen, the higher thromboxane A2 level, and expression of more glycoprotein Ib and IIb/IIIa receptors than smaller platelets [28]. The increased release of proinflammatory cytokines in patients with vitamin D deficiency increases oxidative stress and enables release of immature and activated platelets from the bone marrow, with an increased mean platelet volume [28].

Vitamin D deficiency was associated with coronary heart disease and myocardial infarction [87] and was found in a high proportion of patients with myocardial infarction [9, 88]. Vitamin D status is prognostic for major* postinfarction adverse events*, such as heart failure hospitalizations, recurrent acute myocardial infarction, death [89, 90], or restenosis after percutaneous coronary intervention [11]. A significant, moderate association was found between circulating vitamin D concentration and the risk of all-cause mortality, especially deaths due to coronary disease [6].

Vitamin D level was not associated with the severity of coronary lesions in patients with ST-segment elevation myocardial infarction [88]. On the other hand, the severity of coronary artery stenosis, assessed according to the Gensini score, a validated measure of the angiographic severity of coronary heart disease, was associated with vitamin D deficiency [91].

Arnson et al. examined the effects of short-term vitamin D supplementation on* inflammatory cytokines *after an acute myocardial infarction, reporting a reduction of vascular cell adhesion molecules, C-reactive protein, and interleukin-6, supporting the cardioprotective anti-inflammatory effects of vitamin D on the vascular system [92].

Racial differences have been reported regarding the associations between serum 25-hydroxyvitamin D and the risk of coronary heart disease in a multiethnic community-based cohort of adults without clinical cardiovascular disease. The association of low calcidiol and increased risk of coronary heart disease was demonstrated in white and Chinese study participants, but not in black or Hispanic [93].

Vitamin D exerts biological effects on cardiac myocytes, stimulating calcium-ATPase activity and calcium uptake in cardiac myocytes [11]. Lack of vitamin D could cause diastolic dysfunction, and the Hoorn study found a trend towards increased risk of diastolic dysfunction in persons with vitamin D deficiency, considering 614 persons from a population-based cohort of older men and women [94]. No significant association was found between vitamin D levels and left ventricular diastolic performance, including left atrial volume index in a retrospective observational study including 1,011 unselected patients (involving patients with hypertension and diabetes) [95]. Several explanations were found for the lack of association between vitamin D level and diastolic dysfunction, such as the cross-sectional study design and insufficient information about the duration of vitamin D deficiency [95].

The majority of congestive heart failure patients have insufficient vitamin D, due to reduced sunlight exposure, difficult mobilization and outdoor activity, nutritional factors, and malabsorption of vitamin D due to intestinal edema in severe right heart failure and comorbidities, such as obesity and renal and hepatic failure [10]. Lack of vitamin D causes hypocalcemia and* secondary hyperparathyroidism*. Indeed, serum parathormone (PTH) was elevated in patients with congestive heart failure due to ischemic or dilated cardiomyopathy and hypovitaminosis D was present [96]. Osteomalacia and osteoporosis and fracture rates should be, probably, evaluated in individuals with congestive heart failure [10]. Osteoporosis and cardiovascular pathology share common backgrounds, including osteoprotegerin and receptor activator of nuclear factor kappa-B ligand, involved in osteoclast activation and vascular calcification and atherosclerosis; bone morphogenetic protein, involved in osteoblastic differentiation and atherosclerotic lesions, and age-related estrogen deficiency [97]. An inverse association between PTH and vitamin D level persists until the vitamin D value exceeds 30 ng/mL [72]. The presence of hypocalcemia, osteopenia, or osteomalacia could justify vitamin D supplementation in heart failure patients, despite controversial causative link between vitamin D deficiency and heart failure. An* autocrine/paracrine vitamin D system* also exists, independent of PTH level, besides the endocrine vitamin D system [74].

It is important to determine the vitamin D level in patients with myocardial infarction and correct deficient levels. Vitamin D repletion to prevent cardiac remodeling after a myocardial infarction deserves future study [87]. Vitamin D signaling plays an important cardioprotective role after myocardial infarction through anti-inflammatory,* antifibrotic, and antiapoptotic* mechanisms [98]. Despite several studies revealing vitamin deficiency in congestive heart failure patients, no clear data on improvement of outcome with vitamin D supplementation exist, despite reduction of inflammatory markers and PTH level [10]. The RECORD randomized controlled trial demonstrated that vitamin D supplementation might* protect against cardiac failure* in the elderly, but not against myocardial infarction or stroke [99].

Vitamin D receptor knockout mice had upregulated matrix metalloproteinases, involved in cardiac remodeling, impaired cardiac relaxation and contractility, and developed left ventricular hypertrophy [27, 100]. It seems that both matrix metalloproteinases and inhibitors of metalloproteinases expressions are modulated by vitamin D [100].

Vitamin D decreases fibrosis in mesenchymal multipotent cells through the increased expression and nuclear translocation of vitamin D receptors, decreasing profibrotic factors (transforming growth factor B1 and plasminogen activator inhibitor) and several collagen isoforms and increasing expression of antifibrotic factors [101].

Vitamin D deficiency is associated with an increased prevalence of coronary heart disease with adverse outcomes, considering the proatherogenic and profibrotic effects, impaired coronary perfusion, and cardiac remodeling.

## 8. Vitamin D and Left Ventricular Hypertrophy

Murine models, lacking vitamin D receptor, exhibit increased ventricular mass, higher atrial natriuretic peptides, and impaired homeostasis of metalloproteinases and fibroblasts, leading to ventricular dilatation and impaired electromechanical coupling [2]. Considering* hypertension *associated with low vitamin D levels, left ventricular hypertrophy could also be a consequence. O'Connell et al. demonstrated that calcitriol increases* myocyte protein levels and cell size,* suggesting that it induces cardiac myocyte hypertrophy [33]. Blocking the S phase of the cell cycle is the mechanism by which 1,25(OH)2D3 regulates* myocyte proliferation* [33].

Vitamin D reduces cardiac hypertrophy in spontaneously hypertensive rats [102] and in salt-sensitive rats via modulation of several protein kinase pathways [52, 103]. Among the proposed cardioprotective effects of vitamin D, reduced expression of mediators of myocardial hypertrophy, including atrial natriuretic peptides, and growth factors promoting cell proliferation were mentioned [10].

Intravenous calcitriol treatment, used to control secondary hyperparathyroidism in hemodialysis patients, caused regression of myocardial hypertrophy and reduction of QT interval dispersion, suggesting a cardioprotective effect of vitamin D [104]. The addition of calcitriol to cardiomyocytes inhibits cell proliferation without apoptosis, promoting cardiac differentiation [87]. A significant relationship between vitamin D level and interventricular septum and left ventricular mass index was found after adjusting for age, hypertension, and vitamin D therapy status, in a large retrospective study, suggesting the role of vitamin D in ventricular remodeling [95].* Calcium* is also involved in cellular proliferation and activates AKT, a protein kinase involved in the development of cardiac hypertrophy [105]. Calcium increases after vitamin D supplementation and could also enable cardiac hypertrophy, and calcium overload causes also myocyte apoptosis and cardiac arrhythmias [105].

The results reporting the effect of vitamin D on left ventricular hypertrophy are not convincing, ranging from favorable influences to negative results [106].

## 9. Vitamin D and Atrial Fibrillation

Conflicting results were also found regarding the association of low vitamin status and atrial fibrillation. A relationship between vitamin D deficiency and nonvalvular atrial fibrillation was reported by several studies [107, 108]. Serum 25-hydroxyvitamin D level correlated with the* left atrial diameter, high-sensitive C reactive protein, and pulmonary systolic pressure* and was significantly associated with atrial fibrillation in Chinese patients with nonvalvular persistent atrial fibrillation [108]. Direct* electromechanical effects* on the left atrium were revealed by Hanafy et al. for vitamin D, enabling prevention or termination of atrial fibrillation [109].

Rienstra et al. evaluated 2,930 participants of the Framingham Heart Study during a follow-up period of 9.9 years and found no relation between vitamin D status and incident atrial fibrillation, concluding that vitamin D deficiency does not promote the development of atrial fibrillation [110].

## 10. Vitamin D and Stroke

Epidemiological studies have shown that vitamin D deficiency is an independent risk factor for arterial hypertension and stroke [111]. A recent “umbrella” review stated that the association between high vitamin D level and low stroke risk is possible, but not convincing [112].

Additional* neuroprotective* actions of vitamin D have also been reported [111], which may reduce cognitive impairment in poststroke patients [113], and the neuromuscular and osteoprotective effects may improve mobility. It is premature to recommend vitamin D supplementation for the prevention and treatment of stroke, considering that randomized controlled trials did not confirm that vitamin D reduces stroke incidence [111]. The high prevalence of vitamin D deficiency in patients with hypertension and stroke, associated with musculoskeletal pathology, could justify the evaluation, prevention, and treatment of vitamin deficiency in these patients [111].

## 11. Vitamin D and Peripheral Arterial Disease

Vitamin D receptors may be also found in the vascular wall, suggesting that vitamin D status might play a role in the pathogenesis of arterial disease [114]. Among individuals with peripheral artery disease, low vitamin D status was associated with a* faster decline of functional performance* but not with mortality [115]. Vitamin D deficiency was highly prevalent in patients with occlusive and aneurysmatic arterial disease, independent of traditional cardiovascular risk factors, and showed a strong association with the severity of the arterial disease and atherosclerotic markers:* carotid artery intima-media thickness and ankle-brachial index and high sensitive C reactive protein* [114]. It was suggested that the relationship between low vitamin D status and arterial disease is mediated by an independent arterial wall effect [114]. Severe vitamin D deficiency results in a disrupted adaptive* immune response and an inflammatory milieu,* promoting vascular dysfunction and insulin resistance [2, 116].

Only few studies examined the effects of vitamin D on vascular function and the results are contradictory [2]. Vitamin D also affects* aortic stiffness and vascular aging* [117, 118]. Activation of the RAA system and subsequent synthesis of angiotensin II increase vascular tone and arterial stiffness, preceding the development of hypertension [2]. A study, including 62 diabetic participants, identified no beneficial effects on cardiovascular risk, insulin resistance, and arterial stiffness after 24 weeks of vitamin D supplementation [119]. The lack of reduction in arterial stiffness might be due to the negative effects of vitamin D supplementation on arterial-stiffness related cardiovascular risk factors and the insufficient duration of the therapy [119].

*Vascular calcifications,* the result of calcium-phosphate deposition, major determinants of mortality and morbidity in affected patients, are associated with excessive vitamin D and hyperphosphatemia. Arterial calcifications occur in the vascular intima, associated with atherosclerosis and lipid accumulation, or in the media, associated with arteriosclerosis due to age, diabetes, and end-stage renal failure; both forms increase vascular stiffness [34, 120].

Physiologic vascular vitamin D actions include inhibitions of proatherogenic processes and intimal and medial artery calcification, including release of proinflammatory cytokines and adhesion molecules, migration, and proliferation of vascular smooth muscle cells [121].

## 12. Renal Implications of Vitamin D Deficiency Related to Cardiovascular Pathology

The kidneys are involved in the synthesis of the metabolically active form of vitamin D, since the second hydroxylation, stimulated by the parathyroid hormone, occurs in the kidneys. Serum phosphate levels influence the renal hydroxylation of vitamin D through a negative feedback mechanism [122]. Individuals with renal disease have a deficiency of 1,25-dihydroxyvitamin D, impairing calcium and phosphate balance [122].

Cardiovascular diseases are more prevalent in patients with chronic kidney disease compared to patients with normal kidney function, and several links between vitamin D deficiency and poor cardiovascular outcomes were described in patients with renal disease [43].

Mortality, due mainly to cardiovascular causes, was associated with low vitamin D levels and high parathyroid hormone in patients with chronic renal disease [43]. The randomized Japan Dialysis Active Vitamin D Trial (J-DAVID), with the following primary outcomes: fatal or nonfatal cardiovascular events, coronary interventions, and lower limb artery intervention in hemodialysis patients, will, probably, provide valuable data regarding cardiovascular events in patients with chronic kidney disease stage 5, considering active vitamin D [43].

*Vascular calcifications* were found also in experimental uremic models with low levels of vitamin D [123]. They are associated with an increased cardiovascular mortality in stage 5 chronic kidney disease [120], and renal osteodystrophy and its therapy, the use of warfarin, and, probably, other elements of the uremic milieu may contribute to its etiology [34].

Vitamin D deficient patients with chronic renal failure had* enhanced atherosclerotic lesions* with arterial stiffening and reduced flow-mediated dilatation [97]. Animal studies evaluating the effects of vitamin D compounds on uremic vascular calcifications and pulse wave velocity revealed a dose-response relationship on vascular calcifications and a differential effect of different compounds, suggesting different mechanisms of action [43, 120]. Low doses of calcitriol and paricalcitol, sufficient to correct secondary hyperparathyroidism, were protective against aortic calcification in a mouse model of chronic kidney disease, but higher doses stimulate further calcification [124]. Calciphylaxis, a highly morbid and severe type of vascular calcification, was reported to be more prevalent in patients treated with calcitriol, but not with selective vitamin D analogues [43].

Patients on* chronic dialysis* are at increased risk of vitamin D deficiency, and six months of cholecalciferol therapy did not improve* blood pressure, arterial stiffness, and cardiac function* [125].

*Elevated PTH* contributes probably to the development of* uremic cardiomyopathy*, considering the correlations between PTH and left ventricular hypertrophy in chronic renal failure [126]. Vitamin D given in hemodialysis patients enabled regression of myocardial hypertrophy and reduction of QT interval dispersion, a marker of ventricular arrhythmia risk [104].

*Vitamin D receptor polymorphisms*, such as B alleles of BsmI, with altered vitamin D signaling, are genetic risk factors for the development of left ventricular hypertrophy in kidney disease [127–129]. Left ventricular hypertrophy is a strong cardiovascular risk marker in patients with end-stage renal disease [127–129]. The possible mechanisms responsible for the increased mortality associated with BsmI polymorphism in hemodialysis patients are as follows: modification of vitamin D receptor sensitivity and expression in cardiac and vascular tissues, modification of the circulating levels of vitamin D due to the influence of vitamin D receptors on the feedback mechanism for the regulation of alpha-1-hydroxylase, hyperparathyroidism with calcium-phosphate imbalance, which predisposes to cardiac and vascular calcifications, and hampered calcitriol effects [130].

## 13. Why Conflicting Results?

At present, the data regarding the causal link between low vitamin D status and cardiovascular disease are mixed, conflicting, and ambiguous. Several reasons for conflicting results were found, including significant heterogeneity of vitamin D doses, baseline concentration, therapy duration and compounds, differences of absorption and metabolism among individuals, genetic differences in the vitamin D receptor, private use of vitamin D, biases due to different diseases, study-design related factors, variations in definitions [43], several potentially confounding factors, including age, body mass index, medication, diet, sunlight exposure, physical activity, and concomitant intake of calcium [114, 131, 132], latency of the effect of vitamin D, inappropriate follow-up time or lack of a control group with normal vitamin D level [9], lack of standardization of 25-hydroxyvitamin D assay, different ethnic populations [83], autocrine and paracrine vitamin D systems, local tissue vitamin D intoxication, concomitant hyperphosphatemia, PTH level, and counterregulatory hormones, such as fibroblast growth factor 23 [74, 133]. Assessment of vitamin D status only from dietary questionnaires has, probably, a high degree of subjectivity. Future investigations should focus also on bioavailability rather than total 25-hydroxyvitamin D [43]. A high cardiovascular disease incidence and prevalence were found at high latitudes and geographical areas with low exposure to ultraviolet B radiation [20]. Winter and spring months would probably show higher proportions of patients with vitamin D deficiency. The risk of mortality was significantly higher in studies with lower baseline use of vitamin D [6]. Calcium intake may affect the results, because oral calcium may increase the risk of cardiovascular disease [134]. Most of the studies are observational and they should be replicated in randomized controlled trials [112]. The studies are very different, including observational studies of plasma vitamin D concentrations, cross-sectional, longitudinal, systematic literature reviews, and randomized controlled trials of vitamin D supplementation and experimental studies exploring the mechanisms of the associations. Even meta-analyses of randomized studies may not be convincing, especially due to limited sample and low level of significance. Bias due to selection of participants, comparability of study groups, and selection of outcomes of interest [6] could also contribute to different results.

It is still not clear if vitamin D supplementation is needed only if vitamin D is deficient in order to exert its cardioprotective effects. Which type of vitamin D or vitamin D analogue is effective is another question still requiring an answer. Lower doses in vitamin D2 supplementation and shorter intervention periods were associated with a higher mortality [6].

The question about the benefit of vitamin D supplementation for cardiovascular outcomes cannot be answered certainly for the moment [76], but perhaps the outcomes of the VITAL prevention trial and J-DAVID trial will provide more answers.

Another concern is related to the vitamin D level with benefic effects for cardiovascular disease, considering that doses recommended for osteoporosis treatment are neither beneficial nor harmful in cardiovascular disease [29]. Consumption of high amounts of vitamin D may interfere with the regulation of phosphate metabolism by fibroblast growth factor 23 and the Klotho gene product [133]. It is therefore important to identify and use new markers for phosphate homeostasis, such as salivary phosphate secretion [105], during vitamin D therapy.

It still remains uncertain whether the association between low vitamin D status and cardiovascular diseases is causal or just a bystander. It is likely that unidentified factors and relationships with other endocrine networks are also involved in vitamin D biology, emphasizing the need of further research in this area [74].

## 14. Conclusions

Maintaining an optimal vitamin D serum level seems important not only for calcium homeostasis but also for cardiovascular risk, blood pressure control, prevalence of stroke, metabolic syndrome, and peripheral artery disease. Observational data support the link between vitamin D status and cardiovascular diseases, and vitamin D deficiency can be considered a cardiovascular risk marker. Vitamin D exerts its cardiovascular effects by reducing the activity of the renin-angiotensin-aldosterone system, lowering blood pressure values, and having an anti-inflammatory, antiproliferative, antihypertrophic, antifibrotic, antidiabetic, and antithrombotic effect and beneficial modulation of classical cardiovascular risk factors. The mentioned effects might be very important for public health, considering the high prevalence of vitamin D deficiency, the aging population, and the indoor oriented lifestyle.

Vitamin D deficiency is treatable and supplementation is inexpensive. Vitamin D could be combined with antihypertensive agents in order to control blood pressure, as a simple, inexpensive, and important prophylactic method in order to prevent cardiovascular morbidity, especially in the elderly. Even small gains in prevention are important from a public health perspective. Further proteomics and basic research studies are needed in order to identify the missing pieces in the vitamin D-cardiovascular disease puzzle. Large randomized controlled trials could confirm the promising findings of observational studies, considering endothelial function, arterial stiffness, and patients undergoing percutaneous coronary interventions. Guidelines are needed in order to establish optimal vitamin D level and intake, to maintain a healthy vitamin D status in patients with cardiovascular diseases, and to include vitamin D blood tests, genotyping for vitamin D receptor variants, and serum calcium and phosphates level and bone mineral density as mandatory in evaluating patients with cardiovascular disease. The benefits of screening and treating vitamin D deficiency would be, probably, reflected by reduced cardiovascular morbidity and mortality. Vitamin D supplementation should further consider additional factors, such as phosphates, PTH, RAA, and fibroblast growth factor 23.
